# Biohazard Accidents, Harmful Elements to the Wellness of Healthcare Workers, and Their Risk Factors

**DOI:** 10.3390/ijerph192013214

**Published:** 2022-10-14

**Authors:** Juan José Tejada-Pérez, María Renée Herrera-Burgos, Tesifón Parrón-Carreño, Raquel Alarcón-Rodríguez

**Affiliations:** 1 Faculty of Health Sciences, University of Almeria, Ctra. Sacramento, s/n, 04120 La Cañada, Spain; 2 Occupational Health Service, Poniente Hospital Entrepreneurial Public Health Agency, Ctra. Almerimar, 31, 04700 El Ejido, Spain

**Keywords:** healthcare workers, needlestick injuries, sharp injuries, occupational health, blood-borne pathogens, risk factors

## Abstract

Background. For healthcare personnel, biohazard accidents pose a significant risk to their health. These exposures can enable the transmission of pathogens such as Hepatitis B, Hepatitis C, and human immunodeficiency virus (HIV). Objective. To indicate and quantify the risk associated with higher threatening situations, such as biohazard accidents on repeated occasions or incorrect notifications to injured healthcare professionals. Methods. A cross-sectional study was conducted at the Poniente Hospital in Almeria (Spain). In total, 592 participants reported 1062 accidents and their characteristics and notifications were analyzed. Results. It was found that women (OR = 1.29) working in the surgical area (OR = 2.92), those on indefinite contracts (OR = 1.67), and those with high work experience (OR = 1.14) were the main risk factors for multiple biohazard accidents. Concerning the incorrect notification of these accidents, the main risk factors were work performance during the afternoon shift (OR = 1.72) and the fact that the accident was caused by the injured worker himself (OR = 1.53). Conclusions. This study outlined the main factors that can contribute to healthcare professionals suffering these accidents. As a result, corrective measures must be taken against these risk factors to improve safety for healthcare workers in the future.

## 1. Introduction

Biohazard accidents are considered to be major events due to the serious danger they pose to the wellbeing of healthcare workers. During the performance of their normal tasks, healthcare personnel may be accidentally exposed to potentially contaminated bodily fluids of the patients they take care of [1,2]. The procedures which have shown the greatest exposure risk were those involving the use of sharp or cutting material [3]. Due to the great threat to the welfare of healthcare professionals, needlestick or sharp injuries have traditionally been studied more than splash exposures between body fluids and mucous membranes of healthcare staff [4].

These exposures have been shown to be the source of an increased viral transmission of viruses such as Hepatitis B, Hepatitis C, and human immunodeficiency virus (HIV) [5]. These pathogens can cause serious illnesses such as hepatitis, acquired immunodeficiency syndrome (AIDS) or other complications derived from the abovementioned [6,7]. These potential infections also have a major impact on the economy of the national healthcare systems at all levels [8]. For these reasons, the international community is constantly evaluating the introduction of measures that can help diminish biohazard accidents.

Occupational Health Services (OHS) attempt to reduce the risk of infection by activating their protocols when these events occur. Typically, the injured professional is initially interviewed, preferably during the first 24 h after exposure. These interviews allow the OHS to collect information about the characteristics of the accident, the serological status of the source patient (SSP), and the pre-exposure healthcare professional serology. Using this information, the OHS evaluates the risk of infection, as well as the need to initiate chemoprophylaxis and analytic follow-up of the injured worker [9,10].

Based on research conducted by occupational health departments in different geographical areas around the world, several potential risk factors have been identified for a healthcare provider to suffer a biohazard accident [11,12,13] or to perform an incorrect notification for that matter [14,15,16].

The objectives of this study were (i) to identify the associated risk factors for injured healthcare personnel who suffer more than one biohazard accident during their working life compared with those who only suffer one event during the same period, and (ii) to establish the causes of incorrect notification compared with workers who report their accidents correctly and within the optimal timeframe.

## 2. Material and Methods

### 2.1. Study Design

A cross-sectional epidemiological study was conducted in which cases of biohazard accidents that occurred to healthcare workers were collected, as well as the associated factors that could have influenced the occurrence of these accidents. The data were collected at the Poniente Hospital (Almeria, Southeastern Spain) during the 2001–2018 period.

### 2.2. Study Population

The Poniente Hospital had an average of 1137 healthcare professionals during the study period, with a higher average of women (*n* = 837) than men (*n* = 300). The number of workers who suffered at least one biohazard accident and who participated in our study reporting these accidents totaled 592. These workers reported a total of 1062 accidents.

The inclusion criteria included being a healthcare worker at the hospital, being over 18 years of age, having reported the accident to the OHS, and voluntarily agreeing to participate in the study.

The exclusion criteria included healthcare workers who were not in contact with patients, being under 18 years of age, and/or refusal to participate in the study.

### 2.3. Procedure and Instrumentation

A hospital’s OHS analyses and manages biological accidents that occur to hospital workers. A healthcare worker must report these cases to the OHS within the first 24 h after the incident. Medical staff of the OHS are responsible for interviewing the worker to gather all the details of the accident. After an accident is reported, the SSP is immediately screened for hepatitis B, hepatitis C, and HIV. In the event of a source patient’s positive serology, the injured healthcare personnel’s immunity and serological status are evaluated; in addition, prophylaxis and analytical follow-up are recommended. These actions constitute the hospital’s own protocol for handling biohazard accidents [17].

The variables included in this study were gender, age, professional area, type of contract, shift, work experience, moment of the accident, number of the accident, notification, accident type, material agent, accident liability, serology status before the accident, and knowledge of the identity of the source patient by the injured worker. The information on the accident was stored in the computer program EPINETAC, a Spanish adaptation to the EPINet system (Exposure Prevention Information Network). The main objective of this system is to facilitate the surveillance of and prevention of accidental exposures to blood or biological material in the healthcare environment and to institutionalize a culture of safety within the organization.

### 2.4. Statistical Analysis

A database of the information collected was created. The data were analyzed using IBM SPSS statistical software package (SPSS 26.0 for Windows) [18]. A descriptive analysis of the continuous variables was performed by the calculation of the means and the standard deviations, while for categorical variables, absolute and relative frequency distributions were calculated. Categorical variables were compared using the Chi-square test. The continuous variables were compared using the Mann Whitney U-test, prior to the Kolmogorov–Smirnoff normality test.

A multiple binary logistic regression analysis was used to assess the risk of multiple biohazard accidents and incorrect reporting, adjusting the model with variables that were considered statistically influential based on bivariate analysis. The level of statistical significance was established at a *p* value < 0.05.

### 2.5. Ethical Aspects

Approval for this study was obtained from the Ethics and Research Commission of the Province of Almeria (Spain) (study code: PI_19_16). All procedures were conducted in accordance with the ethical standards of the Declaration of Helsinki. The participation in the study was voluntary. The participants were informed that the data collected based on the EPINETAC questionnaire could be used in future studies and all participants gave their consent.

## 3. Results

There were 1062 biohazard accidents examined, of which 72.1% occurred in female health personnel (*n* = 766/1062). The average age of the participants was 34.96 (±7.9) years, with an average work experience of 7.16 (±5.99) years. This percentage approximated the average percentage of the total female population of the center during the research period, 73.6 % (*n* = 837/1137).

In terms of the number of biohazard accidents suffered by healthcare workers, the characteristics of the workers themselves and the tasks they performed, the healthcare personnel who were involved in multiple biohazard accidents were mainly women with an average age of 36.85 (±7.48) years, previous work experience of 9.71 (±6.14) years, and an indefinite contract. A significant part of this group came from the surgical area (27.6%), handling needles at the time of the accident (29.4%), and also working the morning shift (29.1%). The following information in Table 1 shows the comparison of the working conditions and characteristics of biological hazard accidents between healthcare workers who suffered a single accident and those who suffered multiple. The risk factors which seem statistically significant (*p*-value < 0.05) in relation to the number of biohazard accidents are used later as possible components in our multiple binary logistic regression analysis: age, gender, work experience, professional area, type of contract, work shift, material agent, and moment of the accident.

The notification made by healthcare workers, based on their own personal characteristics, the tasks performed, and the circumstances of accidents revealed that most healthcare staff who incorrectly reported accidents to occupational authorities were women who worked in the surgical area during the afternoon shift (12.4%). In these cases, the healthcare personnel were responsible for injuring themselves (15.1%), and ignoring the serology of the source patient (18.3%). These results are shown in Table 2, which compares the working conditions and the personal characteristics of the workers in the cases where the biohazard accidents were reported correctly and incorrectly. The risk factors that appear as statistically significant (*p*-value < 0.05) in relation to the notification of biohazard accidents are used later as possible components in our multiple binary logistic regression analysis: gender, professional area, work shift, responsible of the accident and knowledge of source serology.

Table 3 shows the results of the multiple binary logistic regression analysis of the factors that may influence the occurrence of multiple biological hazard accidents and incorrect reporting of such accidents.

The model for the multiple accidents of biological risk was adjusted by the following independent variables: age, gender, work experience, type of contract, professional area, work shift, accident type and the type of material with which the accident occurred. A higher risk of having multiple accidents of biological risk was observed in female health workers (OR = 1.29) with indefinite work contracts (OR = 1.67) who work in the surgical area (OR = 2.92). An OR = 1.14 was obtained from the workers’ experience (in years).

For the incorrect notification of the biohazard accident, the model was adjusted by the independent variables: age, gender, worker responsible for the accident, work shift, previous knowledge of the SSP and moment of the accident. Healthcare providers who were responsible for accidental exposure themselves (OR = 1.53) and who worked the afternoon shift (OR = 1.72) were at a higher risk of late notification. However, those professionals who worked during the morning shift had a reduced incorrect notification risk (OR = 0.21).

## 4. Discussion

In this article, needles were the material agent that most biohazard accidents produced, regardless of the number of accidents suffered by each worker. Needlesticks were present in the highest percentages of biohazard accidents compared with other material agents such as surgical equipment. This situation persists in both cases: workers who suffered their first accident, and for those who already had several of them. Our results coincide with those obtained by the research group Bouya et al. These researchers developed a review and meta-analysis of recent scientific literature, concluding that needlesticks potentially contaminated with body fluids currently represent the most prevalent accidental contacts, globally [19,20]. By evaluating the various reasons that may be the cause of this phenomenon, some authors such as Saadeh et al. point out that needles are the most commonly used short-stabbing material by many of the healthcare professional categories, so it is logical to present puncture rates or cuts in these workers [21]. Other research teams point out reasons linked to a misuse of said material (such as the recapping) or malpractice in the actions after its use, as it can be its elimination in an inadequate container or the result of badly planning the handling of this material [22]. In the future, our working group will try to clarify which of these possible causes have contributed to this situation by developing new projects.

The evaluation of the link between gender and the number of biohazard accidents suffered by workers demonstrated that women were at greater risk of suffering more than one accident after the first case, compared with men. These results are consistent with those obtained by research teams such as Hassanipour et al., who stated that women have a higher risk of having these accidents [23]. Some scientific reviews in the field of prevention against various biological agents in health personnel have justified these results by pointing out that female health personnel, like other women from different sectors, may present a higher level of work–family conflict in society compared with men, which leads to higher physical and mental demands that can lead to a greater number of accidents in this gender [24]. This situation has been contrasted in recent years at a general level in the female working population in Spain, so it is possible that it is also applicable to health personnel in this country [25]. However, this result may be due to several factors that are not directly related to the gender of the cases. Some authors, such as Bianco et al., relate this relationship to the fact that nowadays, a large percentage of healthcare workers are women, so it is normal for these accidents to occur more frequently [26]. Other research groups, such as the one led by Garus-Pakowska et al., report that the risk of suffering a greater number of biohazard accidents is related to the performance of healthcare personnel in their nursing tasks. Thus, as women make up a large part of the nursing population, gender would be associated with the number of accidents of biological risk [27]. The causes that may have influenced this association may be due to other unexplained reasons; therefore, the origins of such an outcome should be specified in the future.

After evaluating the risk of suffering multiple biohazard accidents and the relationship with the type of work area, it was found that these results are consistent with those obtained by working groups, such as those of Rapisarda et al., showing a higher incidence in recent years of biohazard accidents in healthcare workers in the surgical area [28]. In pointing out possible causes that justify the results obtained, other authors emphasize that these differences are established by a comparative decrease in the number of needle punctures with surgical equipment after the implementation of safety devices [29]. The fact that the incidence of biohazard accidents with surgical equipment has not significantly decreased compared to needles in the surgery rooms shows that this may be due to the reduced use of safety devices associated with these elements. Dulon et al. suggested that fewer safety devices are being used in surgical equipment [30]; therefore, this situation may have contributed to the higher number of surgical biohazard accidents in our surgical area. Despite the entry of new European legislation regulating the implementation of these mechanisms, the introduction of these safety materials in surgical instruments such as scalpels has been very deficient in most Spanish centers [31]. This situation is applicable to the hospital in this study, since the implementation and training in the correct use of needle safety devices, which began in 2010, has caused a decrease in needle-related accidents in subsequent years. However, these safety accessories have probably not been properly implemented for the surgical material.

When examining the statistically significant relationship found between work experience and the risk of multiple accidents during their working lives, the results of this study are in accordance with those from recent studies which state that healthcare professionals who have 5 or more years of experience may have a higher risk of biohazard accidents. In addition, the cumulative risk of suffering more cases is higher over time as work experience increases [32,33]. On the one hand, this effect can be explained according to the work of Kebede and Gerensea, as the probability of these accidents occurring does not disappear completely over time, even though workers have had an adequate handling of sharp materials and greater practical experience. On the contrary, the accumulated risk is increased by the increasing number of risky procedures that healthcare staff perform throughout their working lives. Other authors, such as Kidoguchi et al., indicate that, after having the first biohazard accident, the work behavior of healthcare staff changes. The healthcare professional who suffers their first biohazard accident develops a process of post-traumatic stress by re-performing similar procedures with which they had the first cases, performing their functions in an inadequate and dangerous way. This predisposes the worker to an increased risk of further accidents [34]. If this were one of the causes, the use of measures to reduce stress in the workplace as well as a better work organization of could reduce the incidence of these events in the future [35].

In addition, the relationship between the permanent contract of a healthcare worker and the possibility of suffering multiple accidents may be due to several factors that have not been thoroughly investigated by the scientific community, such as changes at the psychological level, overconfidence, or workload management [36]. Another possibility is that, as a permanent contract is often granted to healthcare providers with longer work experience, it may become another factor of confusion, having importance and impact on the study variable mentioned before. Further studies are needed to analyze why a worker’s employment situation may adversely affect their safety.

By studying the relationship between the incorrect notification and cause of biohazard accidents, this study presents similar results to those of Bush et al., in which in cases where the worker was the cause of his own accident, it resulted in a risk factor for incorrect reporting of the event, or even failure to do so [36]. These articles explain similar results due to the feelings of the worker, such as shame after suffering an accident and being required to notify the appropriate authorities, or the fear that their skills and attitudes would be undermined or questioned [37]. After pointing out that the feeling of shame is the main cause for late notification of accidents after a biohazard accident, it raises the question whether this cause also extends to the possible underreporting of such cases at the national level. Our research team is currently developing new projects based on these results.

In scientific literature, it is common to find relationships between the shift and the number of biohazard accidents, such as those described by Garus-Pakowska et al. [38]. In our research, we also found a relationship between the shift during which the accidents took place and the number of accidents, finding an association between morning shifts and a higher risk of suffering more events than during afternoon or evening shifts. This can be explained by the increased number of punctures or cutting risk procedures performed in a regulated manner during the morning shift [39]. Contrastingly, the incorrect or incomplete declaration of the event may be due to fatigue or lack of time that the workers may have during the afternoon or night shift [40]. This situation may also arise because of the difficulty of the notification procedure by the affected worker, since the OHS is only operational during the morning shift, which can increase the difficulty for the afternoon shift workers to declare these events, making it easier for those who work in the morning.

### Limitations and Strengths

The main limitation of this study is that probably not all cases of biohazard accidents suffered by healthcare providers during the investigation period were collected. In this scenario, the main causes of this limitation would have been the possible underreporting of cases. New research projects that enable the specification of whether there has been such underreporting in our center are necessary. Among the strengths of this study is the number of cases collected, which is significantly higher than other similar studies. Nevertheless, the possible underreporting of biohazard events would have been largely mitigated by the case collection period, which was more extensive than that of other similar studies.

## 5. Conclusions

This study identified possible risk factors related to biological hazard accidents and the causes of incorrect reporting of such accidents. In the case of the surgical area staff being women, having an indefinite contract, and greater work experience are associated with a higher risk of suffering a greater number of accidents. On the contrary, factors such as the healthcare professional being the cause of the event or the healthcare provider working in the afternoon shift seem to act as factors that predispose affected workers to incorrect accident reporting. Some aspects such as the underreporting rate of these accidents, as well as the causes explaining their gender distribution will be developed in the future through new research articles.

## Figures and Tables

**Table 1:** Association between number of biohazard accidents and possible risk factors.

Variables		Biohazard Accidents		
		First Accident (*n* = 594)	Multiple Accidents (*n* = 468)	*p*
**Age, in years**		33.48 (7.92)	36.85 (7.48)	0.01 ^a^
**Gender**	Man	147 (13.9%)	149 (14.0%)	0.01 ^b^
	Woman	447 (42.1%)	319 (30.0%)	
**Work Experience, in years**		05.15 (5.03)	09.71 (6.14)	0.01 ^a^
**Professional area**	Medical	294 (27.7%)	151 (14.2%)	0.01 ^b^
	Surgical	247 (23.3%)	293 (27.6%)	
	Other	53 (5.0%)	24 (2.2%)	
**Type of contract**	Indefinite	236 (22.2%)	327 (30.8%)	0.01 ^b^
	Temporary	358 (33.7%)	141 (13.3%)	
**Work shift**	Morning	337 (31.7%)	309 (29.1%)	0.01 ^b^
	Afternoon	162 (15.3%)	113 (10.6%)	
	Night	95 (9.0%)	46 (4.3%)	
**Hour Accident**		13.07 (5.26)	12.97 (4.46)	0.63 ^a^
**Material Agent**	Needle	415 (39.1%)	312 (29.4%)	0.01 ^b^
	Surgical	68 (6.4%)	84 (7.9%)	
	Other	111 (10.4%)	72 (6.8%)	
**Moment of the Accident**	During the process	289 (27.2%)	318 (30.0%)	0.01 ^b^
	After the process	305 (28.7%)	150 (14.1%)	
**Responsible**	Worker himself	338 (31.8%)	287 (27.0%)	0.15 ^b^
	Another worker	256 (24.1%)	181 (17.1%)	
**Accident Type**	Percutaneous	496 (46.7%)	401 (37.8%)	0.33 ^b^
	Seromucous	98 (9.2%)	67 (6.3%)	
**Knowledge Source Serology**	Yes	106 (10.0%)	71 (6.6%)	0.25 ^b^
	No	488(46.0%)	397 (37.4%)	

^a^ Associated risk factor variable: Quantitative. Averages (standard deviation) are used as measures of central tendency. ^b^ Associated risk factor variable: Qualitative. Frequency measures (percentages) are used.

**Table 2:** Association between notification and possible risk factors.

Variables		Notification		
		Correct (*n* = 813)	Incorrect (*n* = 249)	*p*
**Age, in years**		35.18 (8.09)	34.24 (7.21)	0.11 ^a^
**Gender**	Man	240 (22.6%)	56 (5.3%)	0.03 ^b^
	Woman	573 (53.9%)	193 (18.2%)	
**Work Experience, in years**		07.21 (6.18)	06.99 (5.35)	0.79 ^a^
**Professional area**	Medical	328 (30.9%)	117 (11.0%)	0.01 ^b^
	Surgical	419 (39.5%)	121 (11.4%)	
	Other	66 (6.2%)	11 (1.0%)	
**Type of contract**	Indefinite	438 (41.2%)	125 (11.8%)	0.31 ^b^
	Temporary	375 (35.3%)	124 (11.7%)	
**Work shift**	Morning	579 (54.5%)	67 (6.3%)	0.01 ^b^
	Afternoon	143 (13.5%)	132 (12.4%)	
	Night	91 (8.6%)	50 (4.7%)	
**Hour Accident**		12.39 (4.43)	15.07 (5.81)	0.16 ^a^
**Material Agent**	Needle	553 (52.0%)	174 (16.4%)	0.68 ^b^
	Surgical	118 (11.1%)	34 (3.2%)	
	Other	142 (13.4%)	41 (3.9%)	
**Moment of the Accident**	During the process	459 (43.2%)	148 (14.0%)	0.50 ^b^
	After the process	354 (33.3%)	101 (9.5%)	
**Responsible**	Worker himself	464 (43.7%)	161 (15.1%)	0.03 ^b^
	Another worker	349 (32.9%)	88 (8.3%)	
**Accident Type**	Percutaneous	688 (64.8%)	209 (19.7%)	0.79 ^b^
	Seromucous	125 (11.8%)	40 (3.7%)	
**Knowledge Source Serology**	Yes	122 (11.5%)	55 (5.1%)	0.01 ^b^
	No	691 (65.1%)	194 (18.3%)	

^a^ Associated risk factor variable: Quantitative. Average (standard deviation) are used as measure of central tendency. ^b^ Associated risk factor variable: Qualitative. Frequency measures (percentages) are used.

**Table 3:** Binary logistic regression analysis for multiple biological hazard accidents and incorrect reporting.

Parameters		OR	95% CI	*p*-Value
**Multiple Accidents**	Professional Area (Surgical Area)	2.92	1.62–5.27	<0.001
	Work Experience	1.14	1.10–1.18	<0.001
	Contract (Indefinite)	1.67	1.20–2.34	<0.01
	Gender (Woman)	1.29	1.06–1.56	<0.05
**Incorrect Reporting**	Cause of the Accident (Worker Themselves)	1.53	1.10–2.11	<0.01
	Shift (Afternoon)	1.72	1.13–2.63	<0.01
	Shift (Morning)	0.21	0.14–0.32	<0.01

## Data Availability

Data of this study are stored in an SPSS software Project.
